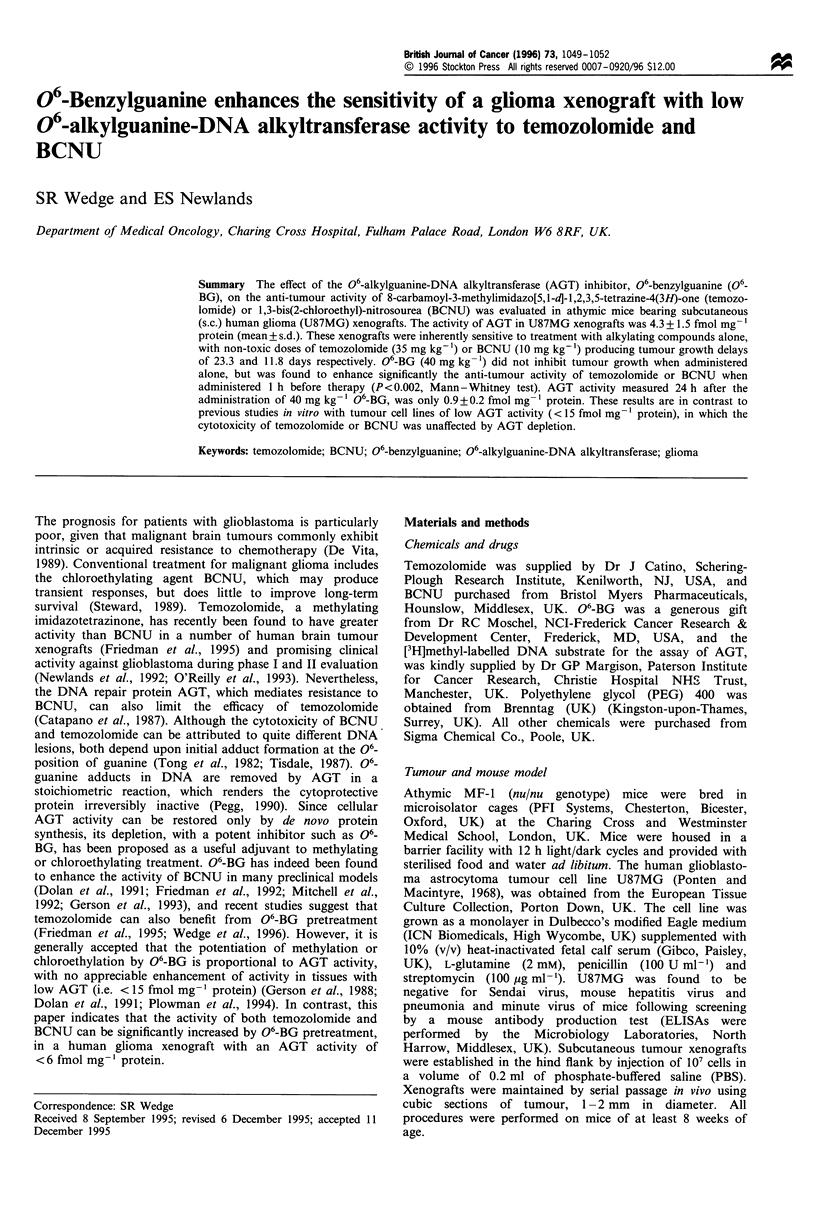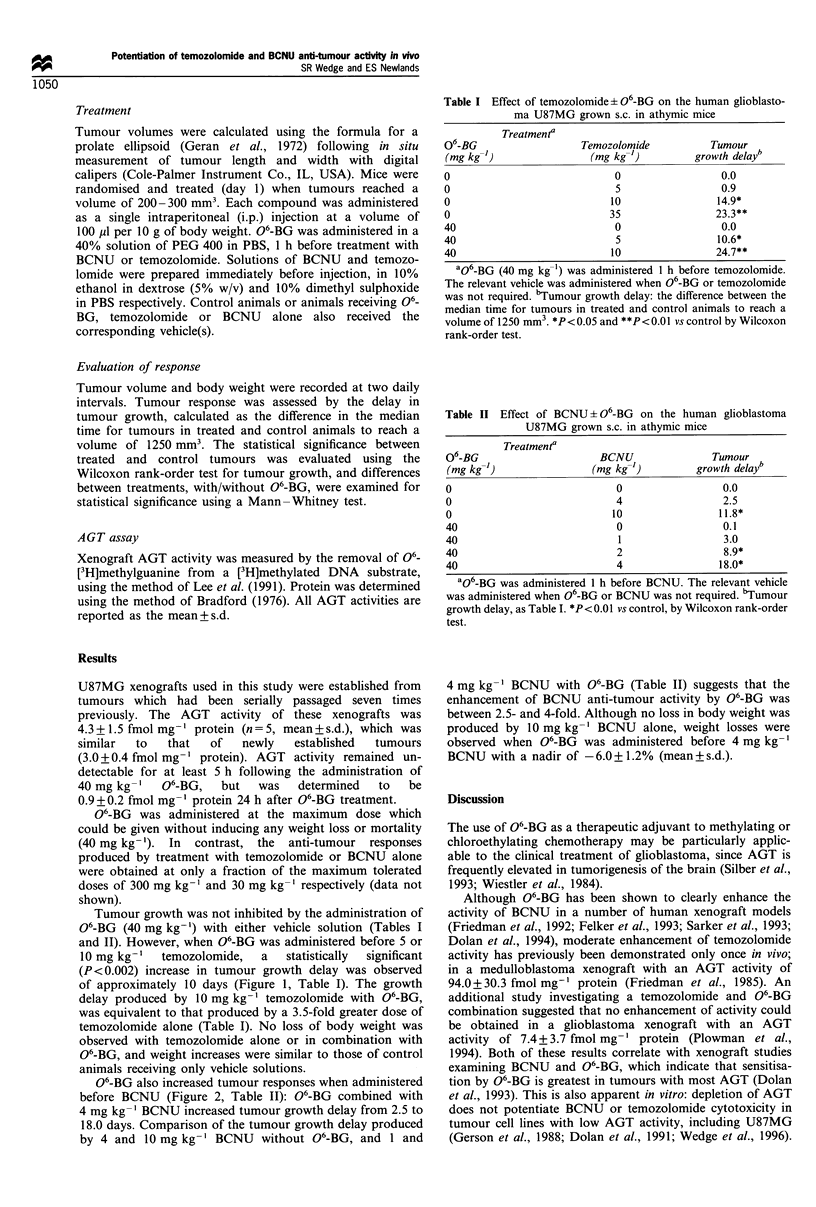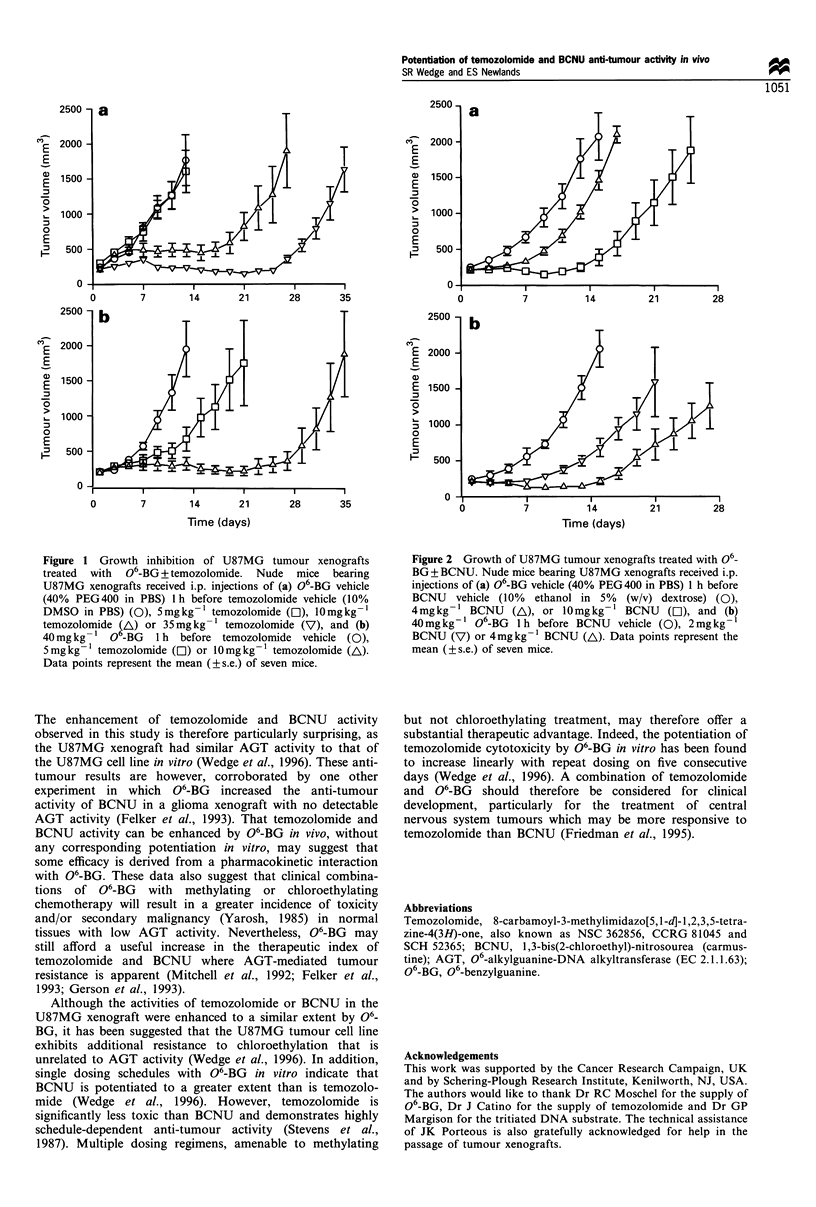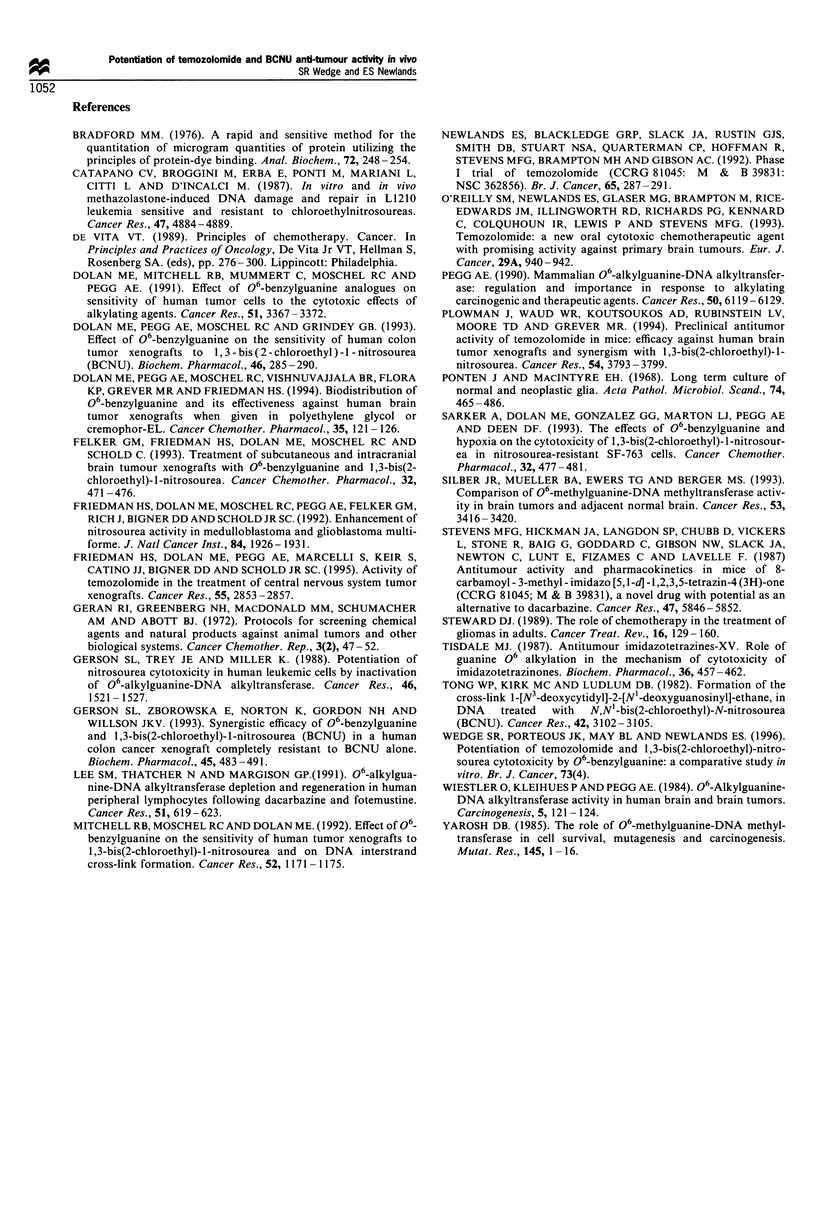# O6-benzylguanine enhances the sensitivity of a glioma xenograft with low O6-alkylguanine-DNA alkyltransferase activity to temozolomide and BCNU.

**DOI:** 10.1038/bjc.1996.203

**Published:** 1996-05

**Authors:** S. R. Wedge, E. S. Newlands

**Affiliations:** Department of Medical Oncology, Charing Cross Hospital, London, UK.

## Abstract

The effect of the O6-alkylguanine-DNA alkyltransferase (AGT) inhibitor, O6-benzylguanine (O6-BG), on the anti-tumour activity of 8-carbamoyl-3-methylimidazo [5,1-d]-1,2,3,5-tetrazine-4(3H)-one (temozolomide) or 1,3-bis(2-chloroethyl)-nitrosourea (BCNU) was evaluated in athymic mice bearing subcutaneous (s.c.) human glioma (U87MG) xenografts. The activity of AGT in U87MG xenografts was 4.3 +/- 1.5 fmol mg-1 protein (mean +/- s.d). These xenografts were inherently sensitive to treatment with alkylating compounds alone, with non-toxic doses of temozolomide (35 mg kg-1) or BCNU (10 mg kg-1) producing tumour growth delays of 23.3 and 11.8 days respectively. O6-BG (40 mg kg-1) did not inhibit tumour growth when administered alone, but was found to enhance significantly the anti-tumour activity of temozolomide or BCNU when administered 1 h before therapy (P < 0.002, Mann-Whitney test). AGT activity measured 24 h after the administration of 40 mg kg-1 O6-BG, was only 0.9 +/- 0.2 fmol mg-1 protein. These results are in contrast to previous studies in vitro with tumour cell lines of low AGT activity (< 15 fmol mg-1 protein), in which the cytotoxicity of temozolomide or BCNU was unaffected by AGT depletion.


British Journal of Cancer (1996) 73, 1049-1052
© 1996 Stockton Press All rights reserved 0007-0920/96 $12.00

### $O^6$-Benzylguanine enhances the sensitivity of a glioma xenograft with low $O^6$-alkylguanine-DNA alkyltransferase activity to temozolomide and BCNU

SR Wedge and ES Newlands

*Department of Medical Oncology, Charing Cross Hospital, Fulham Palace Road, London W6 8RF, UK.*

**Summary** The effect of the $O^6$-alkylguanine-DNA alkyltransferase (AGT) inhibitor, $O^6$-benzylguanine ($O^6$-BG), on the anti-tumour activity of 8-carbamoyl-3-methylimidazo[5,1-*d*]-1,2,3,5-tetrazine-4(3*H*)-one (temozolomide) or 1,3-bis(2-chloroethyl)-nitrosourea (BCNU) was evaluated in athymic mice bearing subcutaneous (s.c.) human glioma (U87MG) xenografts. The activity of AGT in U87MG xenografts was $4.3 \pm 1.5$ fmol $mg^{-1}$ protein (mean ± s.d.). These xenografts were inherently sensitive to treatment with alkylating compounds alone, with non-toxic doses of temozolomide (35 mg $kg^{-1}$) or BCNU (10 mg $kg^{-1}$) producing tumour growth delays of 23.3 and 11.8 days respectively. $O^6$-BG (40 mg $kg^{-1}$) did not inhibit tumour growth when administered alone, but was found to enhance significantly the anti-tumour activity of temozolomide or BCNU when administered 1 h before therapy ($P<0.002$, Mann–Whitney test). AGT activity measured 24 h after the administration of 40 mg $kg^{-1}$ $O^6$-BG, was only $0.9 \pm 0.2$ fmol $mg^{-1}$ protein. These results are in contrast to previous studies *in vitro* with tumour cell lines of low AGT activity (<15 fmol $mg^{-1}$ protein), in which the cytotoxicity of temozolomide or BCNU was unaffected by AGT depletion.

**Keywords:** temozolomide; BCNU; $O^6$-benzylguanine; $O^6$-alkylguanine-DNA alkyltransferase; glioma

The prognosis for patients with glioblastoma is particularly poor, given that malignant brain tumours commonly exhibit intrinsic or acquired resistance to chemotherapy (De Vita, 1989). Conventional treatment for malignant glioma includes the chloroethylating agent BCNU, which may produce transient responses, but does little to improve long-term survival (Steward, 1989). Temozolomide, a methylating imidazotetrazinone, has recently been found to have greater activity than BCNU in a number of human brain tumour xenografts (Friedman *et al.*, 1995) and promising clinical activity against glioblastoma during phase I and II evaluation (Newlands *et al.*, 1992; O'Reilly *et al.*, 1993). Nevertheless, the DNA repair protein AGT, which mediates resistance to BCNU, can also limit the efficacy of temozolomide (Catapano *et al.*, 1987). Although the cytotoxicity of BCNU and temozolomide can be attributed to quite different DNA lesions, both depend upon initial adduct formation at the $O^6$-position of guanine (Tong *et al.*, 1982; Tisdale, 1987). $O^6$-guanine adducts in DNA are removed by AGT in a stoichiometric reaction, which renders the cytoprotective protein irreversibly inactive (Pegg, 1990). Since cellular AGT activity can be restored only by *de novo* protein synthesis, its depletion, with a potent inhibitor such as $O^6$-BG, has been proposed as a useful adjuvant to methylating or chloroethylating treatment. $O^6$-BG has indeed been found to enhance the activity of BCNU in many preclinical models (Dolan *et al.*, 1991; Friedman *et al.*, 1992; Mitchell *et al.*, 1992; Gerson *et al.*, 1993), and recent studies suggest that temozolomide can also benefit from $O^6$-BG pretreatment (Friedman *et al.*, 1995; Wedge *et al.*, 1996). However, it is generally accepted that the potentiation of methylation or chloroethylation by $O^6$-BG is proportional to AGT activity, with no appreciable enhancement of activity in tissues with low AGT (i.e. <15 fmol $mg^{-1}$ protein) (Gerson *et al.*, 1988; Dolan *et al.*, 1991; Plowman *et al.*, 1994). In contrast, this paper indicates that the activity of both temozolomide and BCNU can be significantly increased by $O^6$-BG pretreatment, in a human glioma xenograft with an AGT activity of <6 fmol $mg^{-1}$ protein.

Correspondence: SR Wedge
Received 8 September 1995; revised 6 December 1995; accepted 11 December 1995

### Materials and methods

### *Chemicals and drugs*

Temozolomide was supplied by Dr J Catino, Schering-Plough Research Institute, Kenilworth, NJ, USA, and BCNU purchased from Bristol Myers Pharmaceuticals, Hounslow, Middlesex, UK. $O^6$-BG was a generous gift from Dr RC Moschel, NCI-Frederick Cancer Research & Development Center, Frederick, MD, USA, and the [$^3$H]methyl-labelled DNA substrate for the assay of AGT, was kindly supplied by Dr GP Margison, Paterson Institute for Cancer Research, Christie Hospital NHS Trust, Manchester, UK. Polyethylene glycol (PEG) 400 was obtained from Brenntag (UK) (Kingston-upon-Thames, Surrey, UK). All other chemicals were purchased from Sigma Chemical Co., Poole, UK.

### *Tumour and mouse model*

Athymic MF-1 (*nu/nu* genotype) mice were bred in microisolator cages (PFI Systems, Chesterton, Bicester, Oxford, UK) at the Charing Cross and Westminster Medical School, London, UK. Mice were housed in a barrier facility with 12 h light/dark cycles and provided with sterilised food and water *ad libitum.* The human glioblastoma astrocytoma tumour cell line U87MG (Ponten and Macintyre, 1968), was obtained from the European Tissue Culture Collection, Porton Down, UK. The cell line was grown as a monolayer in Dulbecco's modified Eagle medium (ICN Biomedicals, High Wycombe, UK) supplemented with 10% (v/v) heat-inactivated fetal calf serum (Gibco, Paisley, UK), L-glutamine (2 mM), penicillin (100 U $ml^{-1}$) and streptomycin (100 $\mu g$ $ml^{-1}$). U87MG was found to be negative for Sendai virus, mouse hepatitis virus and pneumonia and minute virus of mice following screening by a mouse antibody production test (ELISAs were performed by the Microbiology Laboratories, North Harrow, Middlesex, UK). Subcutaneous tumour xenografts were established in the hind flank by injection of $10^7$ cells in a volume of 0.2 ml of phosphate-buffered saline (PBS). Xenografts were maintained by serial passage *in vivo* using cubic sections of tumour, 1–2 mm in diameter. All procedures were performed on mice of at least 8 weeks of age.

### *Treatment*

Tumour volumes were calculated using the formula for a prolate ellipsoid (Geran *et al.*, 1972) following *in situ* measurement of tumour length and width with digital calipers (Cole-Palmer Instrument Co., IL, USA). Mice were randomised and treated (day 1) when tumours reached a volume of 200–300 $mm^3$. Each compound was administered as a single intraperitoneal (i.p.) injection at a volume of 100 $\mu l$ per 10 g of body weight. $O^6$-BG was administered in a 40% solution of PEG 400 in PBS, 1 h before treatment with BCNU or temozolomide. Solutions of BCNU and temozolomide were prepared immediately before injection, in 10% ethanol in dextrose (5% w/v) and 10% dimethyl sulphoxide in PBS respectively. Control animals or animals receiving $O^6$-BG, temozolomide or BCNU alone also received the corresponding vehicle(s).

### *Evaluation of response*

Tumour volume and body weight were recorded at two daily intervals. Tumour response was assessed by the delay in tumour growth, calculated as the difference in the median time for tumours in treated and control animals to reach a volume of 1250 $mm^3$. The statistical significance between treated and control tumours was evaluated using the Wilcoxon rank-order test for tumour growth, and differences between treatments, with/without $O^6$-BG, were examined for statistical significance using a Mann–Whitney test.

### *AGT assay*

Xenograft AGT activity was measured by the removal of $O^6$-[$^3$H]methylguanine from a [$^3$H]methylated DNA substrate, using the method of Lee *et al.* (1991). Protein was determined using the method of Bradford (1976). All AGT activities are reported as the mean $\pm$ s.d.

**Table I** Effect of temozolomide $\pm$ $O^6$-BG on the human glioblastoma U87MG grown s.c. in athymic mice

| *Treatment*[a] $O^6$-*BG* (*mg kg*$^{-1}$) | *Temozolomide* (*mg kg*$^{-1}$) | *Tumour growth delay*[b] |
|---|---|---|
| 0 | 0 | 0.0 |
| 0 | 5 | 0.9 |
| 0 | 10 | 14.9* |
| 0 | 35 | 23.3** |
| 40 | 0 | 0.0 |
| 40 | 5 | 10.6* |
| 40 | 10 | 24.7** |

[a]$O^6$-BG (40 mg kg$^{-1}$) was administered 1 h before temozolomide. The relevant vehicle was administered when $O^6$-BG or temozolomide was not required. [b]Tumour growth delay: the difference between the median time for tumours in treated and control animals to reach a volume of 1250 $mm^3$. *$P<0.05$ and **$P<0.01$ *vs* control by Wilcoxon rank-order test.

**Table II** Effect of BCNU $\pm$ $O^6$-BG on the human glioblastoma U87MG grown s.c. in athymic mice

| *Treatment*[a] $O^6$-*BG* (*mg kg*$^{-1}$) | *BCNU* (*mg kg*$^{-1}$) | *Tumour growth delay*[b] |
|---|---|---|
| 0 | 0 | 0.0 |
| 0 | 4 | 2.5 |
| 0 | 10 | 11.8* |
| 40 | 0 | 0.1 |
| 40 | 1 | 3.0 |
| 40 | 2 | 8.9* |
| 40 | 4 | 18.0* |

[a]$O^6$-BG was administered 1 h before BCNU. The relevant vehicle was administered when $O^6$-BG or BCNU was not required. [b]Tumour growth delay, as Table I. *$P<0.01$ *vs* control, by Wilcoxon rank-order test.

### Results

U87MG xenografts used in this study were established from tumours which had been serially passaged seven times previously. The AGT activity of these xenografts was $4.3 \pm 1.5$ fmol $mg^{-1}$ protein ($n=5$, mean $\pm$ s.d.), which was similar to that of newly established tumours ($3.0 \pm 0.4$ fmol $mg^{-1}$ protein). AGT activity remained undetectable for at least 5 h following the administration of 40 mg $kg^{-1}$ $O^6$-BG, but was determined to be $0.9 \pm 0.2$ fmol $mg^{-1}$ protein 24 h after $O^6$-BG treatment.

$O^6$-BG was administered at the maximum dose which could be given without inducing any weight loss or mortality (40 mg $kg^{-1}$). In contrast, the anti-tumour responses produced by treatment with temozolomide or BCNU alone were obtained at only a fraction of the maximum tolerated doses of 300 mg $kg^{-1}$ and 30 mg $kg^{-1}$ respectively (data not shown).

Tumour growth was not inhibited by the administration of $O^6$-BG (40 mg $kg^{-1}$) with either vehicle solution (Tables I and II). However, when $O^6$-BG was administered before 5 or 10 mg $kg^{-1}$ temozolomide, a statistically significant ($P<0.002$) increase in tumour growth delay was observed of approximately 10 days (Figure 1, Table I). The growth delay produced by 10 mg $kg^{-1}$ temozolomide with $O^6$-BG, was equivalent to that produced by a 3.5-fold greater dose of temozolomide alone (Table I). No loss of body weight was observed with temozolomide alone or in combination with $O^6$-BG, and weight increases were similar to those of control animals receiving only vehicle solutions.

$O^6$-BG also increased tumour responses when administered before BCNU (Figure 2, Table II): $O^6$-BG combined with 4 mg $kg^{-1}$ BCNU increased tumour growth delay from 2.5 to 18.0 days. Comparison of the tumour growth delay produced by 4 and 10 mg $kg^{-1}$ BCNU without $O^6$-BG, and 1 and 4 mg $kg^{-1}$ BCNU with $O^6$-BG (Table II) suggests that the enhancement of BCNU anti-tumour activity by $O^6$-BG was between 2.5- and 4-fold. Although no loss in body weight was produced by 10 mg $kg^{-1}$ BCNU alone, weight losses were observed when $O^6$-BG was administered before 4 mg $kg^{-1}$ BCNU with a nadir of $-6.0 \pm 1.2\%$ (mean $\pm$ s.d.).

### Discussion

The use of $O^6$-BG as a therapeutic adjuvant to methylating or chloroethylating chemotherapy may be particularly applicable to the clinical treatment of glioblastoma, since AGT is frequently elevated in tumorigenesis of the brain (Silber *et al.*, 1993; Wiestler *et al.*, 1984).

Although $O^6$-BG has been shown to clearly enhance the activity of BCNU in a number of human xenograft models (Friedman *et al.*, 1992; Felker *et al.*, 1993; Sarker *et al.*, 1993; Dolan *et al.*, 1994), moderate enhancement of temozolomide activity has previously been demonstrated only once *in vivo*; in a medulloblastoma xenograft with an AGT activity of $94.0 \pm 30.3$ fmol $mg^{-1}$ protein (Friedman *et al.*, 1985). An additional study investigating a temozolomide and $O^6$-BG combination suggested that no enhancement of activity could be obtained in a glioblastoma xenograft with an AGT activity of $7.4 \pm 3.7$ fmol $mg^{-1}$ protein (Plowman *et al.*, 1994). Both of these results correlate with xenograft studies examining BCNU and $O^6$-BG, which indicate that sensitisation by $O^6$-BG is greatest in tumours with most AGT (Dolan *et al.*, 1993). This is also apparent *in vitro*: depletion of AGT does not potentiate BCNU or temozolomide cytotoxicity in tumour cell lines with low AGT activity, including U87MG (Gerson *et al.*, 1988; Dolan *et al.*, 1991; Wedge *et al.*, 1996).

**Figure 1** Growth inhibition of U87MG tumour xenografts treated with $O^6$-BG±temozolomide. Nude mice bearing U87MG xenografts received i.p. injections of (**a**) $O^6$-BG vehicle (40% PEG400 in PBS) 1 h before temozolomide vehicle (10% DMSO in PBS) (○), 5 mg kg$^{-1}$ temozolomide (□), 10 mg kg$^{-1}$ temozolomide (△) or 35 mg kg$^{-1}$ temozolomide (▽), and (**b**) 40 mg kg$^{-1}$ $O^6$-BG 1 h before temozolomide vehicle (○), 5 mg kg$^{-1}$ temozolomide (□) or 10 mg kg$^{-1}$ temozolomide (△). Data points represent the mean (±s.e.) of seven mice.

**Figure 2** Growth of U87MG tumour xenografts treated with $O^6$-BG±BCNU. Nude mice bearing U87MG xenografts received i.p. injections of (**a**) $O^6$-BG vehicle (40% PEG400 in PBS) 1 h before BCNU vehicle (10% ethanol in 5% (w/v) dextrose) (○), 4 mg kg$^{-1}$ BCNU (△), or 10 mg kg$^{-1}$ BCNU (□), and (**b**) 40 mg kg$^{-1}$ $O^6$-BG 1 h before BCNU vehicle (○), 2 mg kg$^{-1}$ BCNU (▽) or 4 mg kg$^{-1}$ BCNU (△). Data points represent the mean (±s.e.) of seven mice.

The enhancement of temozolomide and BCNU activity observed in this study is therefore particularly surprising, as the U87MG xenograft had similar AGT activity to that of the U87MG cell line *in vitro* (Wedge *et al.*, 1996). These anti-tumour results are however, corroborated by one other experiment in which $O^6$-BG increased the anti-tumour activity of BCNU in a glioma xenograft with no detectable AGT activity (Felker *et al.*, 1993). That temozolomide and BCNU activity can be enhanced by $O^6$-BG *in vivo*, without any corresponding potentiation *in vitro*, may suggest that some efficacy is derived from a pharmacokinetic interaction with $O^6$-BG. These data also suggest that clinical combinations of $O^6$-BG with methylating or chloroethylating chemotherapy will result in a greater incidence of toxicity and/or secondary malignancy (Yarosh, 1985) in normal tissues with low AGT activity. Nevertheless, $O^6$-BG may still afford a useful increase in the therapeutic index of temozolomide and BCNU where AGT-mediated tumour resistance is apparent (Mitchell *et al.*, 1992; Felker *et al.*, 1993; Gerson *et al.*, 1993).

Although the activities of temozolomide or BCNU in the U87MG xenograft were enhanced to a similar extent by $O^6$-BG, it has been suggested that the U87MG tumour cell line exhibits additional resistance to chloroethylation that is unrelated to AGT activity (Wedge *et al.*, 1996). In addition, single dosing schedules with $O^6$-BG *in vitro* indicate that BCNU is potentiated to a greater extent than is temozolomide (Wedge *et al.*, 1996). However, temozolomide is significantly less toxic than BCNU and demonstrates highly schedule-dependent anti-tumour activity (Stevens *et al.*, 1987). Multiple dosing regimens, amenable to methylating but not chloroethylating treatment, may therefore offer a substantial therapeutic advantage. Indeed, the potentiation of temozolomide cytotoxicity by $O^6$-BG *in vitro* has been found to increase linearly with repeat dosing on five consecutive days (Wedge *et al.*, 1996). A combination of temozolomide and $O^6$-BG should therefore be considered for clinical development, particularly for the treatment of central nervous system tumours which may be more responsive to temozolomide than BCNU (Friedman *et al.*, 1995).

### Abbreviations

Temozolomide, 8-carbamoyl-3-methylimidazo[5,1-*d*]-1,2,3,5-tetrazine-4(3*H*)-one, also known as NSC 362856, CCRG 81045 and SCH 52365; BCNU, 1,3-bis(2-chloroethyl)-nitrosourea (carmustine); AGT, $O^6$-alkylguanine-DNA alkyltransferase (EC 2.1.1.63); $O^6$-BG, $O^6$-benzylguanine.

### Acknowledgements

This work was supported by the Cancer Research Campaign, UK and by Schering-Plough Research Institute, Kenilworth, NJ, USA. The authors would like to thank Dr RC Moschel for the supply of $O^6$-BG, Dr J Catino for the supply of temozolomide and Dr GP Margison for the tritiated DNA substrate. The technical assistance of JK Porteous is also gratefully acknowledged for help in the passage of tumour xenografts.

### References

BRADFORD MM. (1976). A rapid and sensitive method for the quantitation of microgram quantities of protein utilizing the principles of protein-dye binding. *Anal. Biochem.*, **72,** 248–254.

CATAPANO CV, BROGGINI M, ERBA E, PONTI M, MARIANI L, CITTI L AND D'INCALCI M. (1987). *In vitro* and *in vivo* methazolastone-induced DNA damage and repair in L1210 leukemia sensitive and resistant to chloroethylnitrosoureas. *Cancer Res.*, **47,** 4884–4889.

DE VITA VT. (1989). Principles of chemotherapy. Cancer. In *Principles and Practices of Oncology*, De Vita Jr VT, Hellman S, Rosenberg SA. (eds), pp. 276–300. Lippincott: Philadelphia.

DOLAN ME, MITCHELL RB, MUMMERT C, MOSCHEL RC AND PEGG AE. (1991). Effect of $O^6$-benzylguanine analogues on sensitivity of human tumor cells to the cytotoxic effects of alkylating agents. *Cancer Res.*, **51,** 3367–3372.

DOLAN ME, PEGG AE, MOSCHEL RC AND GRINDEY GB. (1993). Effect of $O^6$-benzylguanine on the sensitivity of human colon tumor xenografts to 1,3-bis(2-chloroethyl)-1-nitrosourea (BCNU). *Biochem. Pharmacol.*, **46,** 285–290.

DOLAN ME, PEGG AE, MOSCHEL RC, VISHNUVAJJALA BR, FLORA KP, GREVER MR AND FRIEDMAN HS. (1994). Biodistribution of $O^6$-benzylguanine and its effectiveness against human brain tumor xenografts when given in polyethylene glycol or cremophor-EL. *Cancer Chemother. Pharmacol.*, **35,** 121–126.

FELKER GM, FRIEDMAN HS, DOLAN ME, MOSCHEL RC AND SCHOLD C. (1993). Treatment of subcutaneous and intracranial brain tumour xenografts with $O^6$-benzylguanine and 1,3-bis(2-chloroethyl)-1-nitrosourea. *Cancer Chemother. Pharmacol.*, **32,** 471–476.

FRIEDMAN HS, DOLAN ME, MOSCHEL RC, PEGG AE, FELKER GM, RICH J, BIGNER DD AND SCHOLD JR SC. (1992). Enhancement of nitrosourea activity in medulloblastoma and glioblastoma multiforme. *J. Natl Cancer Inst.*, **84,** 1926–1931.

FRIEDMAN HS, DOLAN ME, PEGG AE, MARCELLI S, KEIR S, CATINO JJ, BIGNER DD AND SCHOLD JR SC. (1995). Activity of temozolomide in the treatment of central nervous system tumor xenografts. *Cancer Res.*, **55,** 2853–2857.

GERAN RI, GREENBERG NH, MACDONALD MM, SCHUMACHER AM AND ABOTT BJ. (1972). Protocols for screening chemical agents and natural products against animal tumors and other biological systems. *Cancer Chemother. Rep.*, **3(2),** 47–52.

GERSON SL, TREY JE AND MILLER K. (1988). Potentiation of nitrosourea cytotoxicity in human leukemic cells by inactivation of $O^6$-alkylguanine-DNA alkyltransferase. *Cancer Res.*, **46,** 1521–1527.

GERSON SL, ZBOROWSKA E, NORTON K, GORDON NH AND WILLSON JKV. (1993). Synergistic efficacy of $O^6$-benzylguanine and 1,3-bis(2-chloroethyl)-1-nitrosourea (BCNU) in a human colon cancer xenograft completely resistant to BCNU alone. *Biochem. Pharmacol.*, **45,** 483–491.

LEE SM, THATCHER N AND MARGISON GP.(1991). $O^6$-alkylguanine-DNA alkyltransferase depletion and regeneration in human peripheral lymphocytes following dacarbazine and fotemustine. *Cancer Res.*, **51,** 619–623.

MITCHELL RB, MOSCHEL RC AND DOLAN ME. (1992). Effect of $O^6$-benzylguanine on the sensitivity of human tumor xenografts to 1,3-bis(2-chloroethyl)-1-nitrosourea and on DNA interstrand cross-link formation. *Cancer Res.*, **52,** 1171–1175.

NEWLANDS ES, BLACKLEDGE GRP, SLACK JA, RUSTIN GJS, SMITH DB, STUART NSA, QUARTERMAN CP, HOFFMAN R, STEVENS MFG, BRAMPTON MH AND GIBSON AC. (1992). Phase I trial of temozolomide (CCRG 81045: M & B 39831: NSC 362856). *Br. J. Cancer*, **65,** 287–291.

O'REILLY SM, NEWLANDS ES, GLASER MG, BRAMPTON M, RICE-EDWARDS JM, ILLINGWORTH RD, RICHARDS PG, KENNARD C, COLQUHOUN IR, LEWIS P AND STEVENS MFG. (1993). Temozolomide: a new oral cytotoxic chemotherapeutic agent with promising activity against primary brain tumours. *Eur. J. Cancer*, **29A,** 940–942.

PEGG AE. (1990). Mammalian $O^6$-alkylguanine-DNA alkyltransferase: regulation and importance in response to alkylating carcinogenic and therapeutic agents. *Cancer Res.*, **50,** 6119–6129.

PLOWMAN J, WAUD WR, KOUTSOUKOS AD, RUBINSTEIN LV, MOORE TD AND GREVER MR. (1994). Preclinical antitumor activity of temozolomide in mice: efficacy against human brain tumor xenografts and synergism with 1,3-bis(2-chloroethyl)-1-nitrosourea. *Cancer Res.*, **54,** 3793–3799.

PONTEN J AND MACINTYRE EH. (1968). Long term culture of normal and neoplastic glia. *Acta Pathol. Microbiol. Scand.*, **74,** 465–486.

SARKER A, DOLAN ME, GONZALEZ GG, MARTON LJ, PEGG AE AND DEEN DF. (1993). The effects of $O^6$-benzylguanine and hypoxia on the cytotoxicity of 1,3-bis(2-chloroethyl)-1-nitrosourea in nitrosourea-resistant SF-763 cells. *Cancer Chemother. Pharmacol.*, **32,** 477–481.

SILBER JR, MUELLER BA, EWERS TG AND BERGER MS. (1993). Comparison of $O^6$-methylguanine-DNA methyltransferase activity in brain tumors and adjacent normal brain. *Cancer Res.*, **53,** 3416–3420.

STEVENS MFG, HICKMAN JA, LANGDON SP, CHUBB D, VICKERS L, STONE R, BAIG G, GODDARD C, GIBSON NW, SLACK JA, NEWTON C, LUNT E, FIZAMES C AND LAVELLE F. (1987) Antitumour activity and pharmacokinetics in mice of 8-carbamoyl-3-methyl-imidazo[5,1-*d*]-1,2,3,5-tetrazin-4(3H)-one (CCRG 81045; M & B 39831), a novel drug with potential as an alternative to dacarbazine. *Cancer Res.*, **47,** 5846–5852.

STEWARD DJ. (1989). The role of chemotherapy in the treatment of gliomas in adults. *Cancer Treat. Rev.*, **16,** 129–160.

TISDALE MJ. (1987). Antitumour imidazotetrazines-XV. Role of guanine $O^6$ alkylation in the mechanism of cytotoxicity of imidazotetrazinones. *Biochem. Pharmacol.*, **36,** 457–462.

TONG WP, KIRK MC AND LUDLUM DB. (1982). Formation of the cross-link 1-[$N^3$-deoxycytidyl]-2-[$N^1$-deoxyguanosinyl]-ethane, in DNA treated with $N,N^1$-bis(2-chloroethyl)-$N$-nitrosourea (BCNU). *Cancer Res.*, **42,** 3102–3105.

WEDGE SR, PORTEOUS JK, MAY BL AND NEWLANDS ES. (1996). Potentiation of temozolomide and 1,3-bis(2-chloroethyl)-nitrosourea cytotoxicity by $O^6$-benzylguanine: a comparative study *in vitro*. *Br. J. Cancer*, **73**(4).

WIESTLER O, KLEIHUES P AND PEGG AE. (1984). $O^6$-Alkylguanine-DNA alkyltransferase activity in human brain and brain tumors. *Carcinogenesis*, **5,** 121–124.

YAROSH DB. (1985). The role of $O^6$-methylguanine-DNA methyltransferase in cell survival, mutagenesis and carcinogenesis. *Mutat. Res.*, **145,** 1–16.